# Heterogeneity of lymphoid cells in PBMCs in the acute phase of SFTS: Single-cell transcriptome profiling

**DOI:** 10.7555/JBR.39.20250250

**Published:** 2026-03-19

**Authors:** Jiaying Zhao, Ruowei Xu, Tingting Zhou, Ke Jin, Jin Zhu, Jinhai Zhang, Yifang Han, Xinjian Liu, Dafeng Lu, Chunfang Wang, Jiaojiao Qian, Chunhui Wang, Jun Li

**Affiliations:** 1 Department of Infectious Diseases, the First Affiliated Hospital of Nanjing Medical University, Jiangsu Province Hospital, Nanjing, Jiangsu 210029, China; 2 Epidemiological Department, Huadong Medical Institute of Biotechniques, Nanjing, Jiangsu 210002, China; 3 School of Life Sciences, Nanjing Normal University, Nanjing, Jiangsu 210046, China; 4 School of Basic Medical Sciences, Nanjing Medical University, Nanjing, Jiangsu 211166, China; 5 School of Public Health, Nanjing Medical University, Nanjing, Jiangsu 211166, China

**Keywords:** thrombocytopenia syndrome, *Dabie bandavirus*, single-cell RNA sequencing, lymphoid cells, glucocorticoid

## Abstract

Severe fever with thrombocytopenia syndrome (SFTS), caused by *Dabie bandavirus* (DBV), triggers aberrant immune activation and cytokine storms, contributing to poor prognosis; however, its immune dysfunction mechanism remains unclear. Current management relies on symptomatic treatment and glucocorticoids, but no standardized treatment guidelines exist. This study investigated the mechanisms of abnormal lymphocyte function in acute-phase SFTS and the effects of glucocorticoid treatment on lymphoid cells using single-cell RNA sequencing (scRNA-seq) and bioinformatics analysis. We enrolled three healthy volunteers and 13 patients with acute SFTS and divided them into four groups. ScRNA-seq was performed on peripheral blood mononuclear cells from all 16 participants, capturing transcripts from the 3′ ends of mRNA. Bioinformatics analyses were used to profile patient immunological signatures, characterize subpopulation compositions, infer developmental trajectories, and assess lymphoid cell interactions. We obtained 120886 lymphoid cells, which were clustered into 23 functionally heterogeneous subsets. Results showed that patients with severe SFTS exhibited stronger inflammatory and adaptive immune responses. Glucocorticoid treatment suppressed inflammation and the interferon response but also inhibited the production of virus-specific antibodies. These findings suggest that appropriate glucocorticoid administration may alleviate the hyperinflammatory state in severe SFTS during the acute phase, although it is not recommended as a conventional treatment because of its potential to suppress antiviral immunity. This study provides insights into SFTS immunopathology and informs the optimized clinical use of glucocorticoids.

## Introduction

Severe fever with thrombocytopenia syndrome (SFTS) is a naturally occurring epidemic disease caused by *Dabie bandavirus* (DBV) infection, also known as SFTS bunyavirus^[1]^. This emerging tick-borne infection was first identified in rural China in 2009 and has since become predominantly endemic in East Asia, with increasing reports from China, the Republic of Korea, Japan, and more recently in Southeast Asia^[2]^. Clinically, SFTS is primarily characterized by fever, thrombocytopenia, leukopenia, and gastrointestinal symptoms^[3]^. Severe cases may progress to neuropsychiatric manifestations or life-threatening complications, such as shock, respiratory failure, disseminated intravascular coagulation, and multiple organ dysfunction syndrome, which can be fatal^[2–3]^. Based on disease severity, SFTS is divided into four types: mild, ordinary, severe, and critical^[4]^. The ordinary and severe forms account for approximately 85% of cases, while the mild and critical types constitute approximately 15%^[4]^. The overall mortality rate ranges from 12% to 50%^[5–6]^. Moreover, 19.1%–37.4% of SFTS patients develop central nervous system involvement, and among those with encephalitis or encephalopathy, the mortality rate is approximately 40%^[7–8]^. Given its wide geographic distribution, high risk of severe progression, and substantial mortality, the World Health Organization (WHO) has listed SFTS as a highest-priority disease in the Research and Development Blueprint since 2017^[2]^.

The poor prognosis of SFTS patients is primarily driven by high viral load, cytokine storms, and impaired immune responses resulting from humoral immunity dysfunction^[9]^. However, the exact mechanisms of SFTS pathogenesis remain incompletely understood. Emerging evidence suggests that DBV-induced lymphocytic impairment, characterized by quantitative depletion, qualitative dysfunction, and excessive cytokine release, exacerbates disease severity by disrupting adaptive immunity^[2]^. Upon infection, DBV triggers antigen presentation and cytokine-mediated adaptive immune responses. However, early T cell exhaustion and defective B cell maturation in SFTS patients severely compromise immune defense. Studies have reported a progressive decline in CD4^+^ T cell counts that correlates with disease severity during the critical phase^[10]^. Notably, SFTS patients exhibit elevated expression of apoptotic markers (annexin Ⅴ and CD95) on both CD4^+^ and CD8^+^ T cells, indicating dysregulated lymphocyte survival^[11]^. In humoral immunity, DBV infection disrupts B cell homeostasis, as evidenced by expanded plasmablasts and impaired IgG class-switching^[12]^. Although T/B cell imbalance and cytokine storms are recognized contributors to SFTS progression, the precise mechanisms of DBV-induced lymphocyte dysfunction warrant further investigation^[12–13]^.

The management of SFTS remains clinically challenging due to unresolved aspects of DBV pathogenesis, particularly the poorly defined virus-host immune dynamics, which underlie the lack of targeted therapies or effective vaccines^[2]^. Current treatment primarily relies on supportive care, plasma exchange, intravenous immunoglobulin, and empirical antiviral agents (*e.g.*, arbidol, favipiravir, ribavirin). Although glucocorticoids can help control severe inflammation, they present a therapeutic paradox by potentially impairing antiviral immunity^[3]^. Their precise immunomodulatory effects in SFTS remain unclear, contributing to the continued absence of standardized treatment protocols.

Therefore, we employed high-resolution single-cell RNA sequencing (scRNA-seq) to comprehensively characterize the transcriptomic profiles of lymphoid cells in peripheral blood mononuclear cells (PBMCs) from patients with acute-phase SFTS. This approach enabled us to preliminarily elucidate the mechanisms underlying lymphoid cell-mediated immune dysregulation and to evaluate the immunomodulatory effects of glucocorticoids on these cells.

## Materials and methods

### Study participants

**Figure 1:**
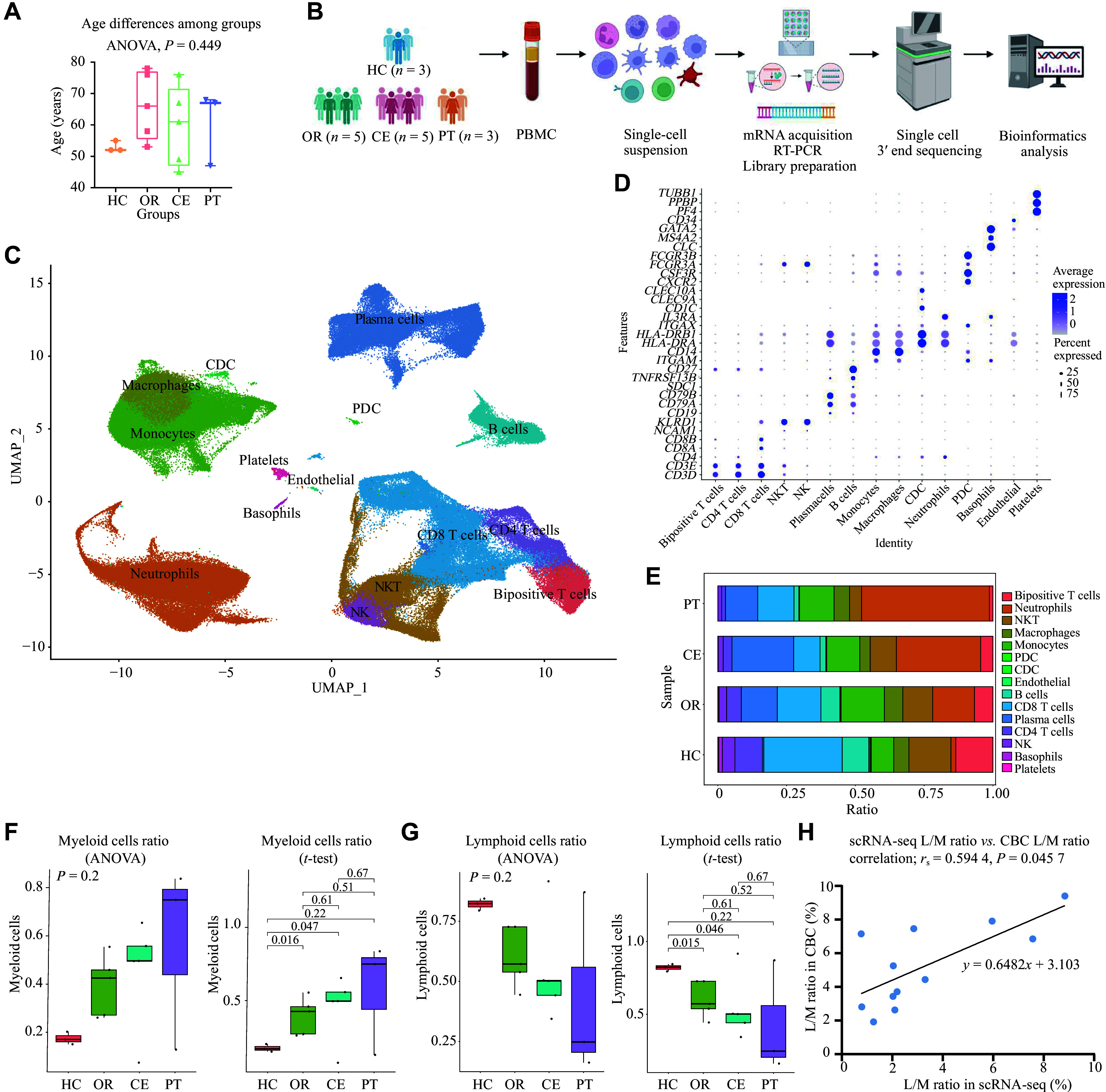
Single-cell transcriptomic immune profiling of peripheral blood mononuclear cells (PBMCs) in the acute phase of severe fever with thrombocytopenia syndrome (SFTS) by single-cell RNA sequencing (scRNA-seq). A: Box plot showing the distribution of age among HC, OR, CE, and PT groups (*P* > 0.05; one-way ANOVA). B: Experimental workflow: (i) Human PBMCs were isolated from peripheral blood; (ii) mRNA captured by barcoding beads was reverse-transcribed into cDNA and used for library preparation; and (iii) scRNA-seq analysis was performed (created with biorender.com). C: UMAP analysis identified 15 major clusters. Data represent *n* = 214726 cells from *n* = 16 biologically independent samples. D: Dot plot depicting expression of canonical gene markers used to identify 15 immune-related cell clusters in PBMCs. E: Bar chart depicting the composition of myeloid and lymphoid cells in patients with SFTS across HC, OR, CE, and PT groups. F and G: Box plots showing the ratio of myeloid and lymphoid cells in PBMCs among patients in HC, OR, CE, and PT groups. Statistical analysis was performed using ANOVA and two-tailed Student's *t*-tests. Boxes represent median ± interquartile range (IQR). H: Bivariate correlation analysis between CBC-derived L/M ratio and scRNA-seq-quantified L/M ratio in 12 peripheral blood samples from the SFTS patients enrolled in scRNA-seq. Abbreviations: CBC, complete blood count; CDC, conventional dendritic cells; CE, severe cases without glucocorticoid therapy; HC, healthy controls; L/M, ratio of lymphocytes to monocytes; NK, natural killer cells; NKT, natural killer T cells; OR, ordinary cases; PDC, plasmacytoid dendritic cells; PT, severe cases receiving glucocorticoid therapy; UMAP, uniform manifold approximation and projection.

In this study, we performed scRNA-seq on PBMC samples from five ordinary SFTS cases (OR group, Matrix4–Matrix8), five severe cases without glucocorticoid therapy (CE group, Matrix9–Matrix13), and three severe cases receiving glucocorticoid therapy (PT group, Matrix14–Matrix16), all diagnosed at the First Affiliated Hospital of Nanjing Medical University between June and October, 2022. Three healthy subjects (HC group, Matrix1–Matrix3) were enrolled as age- and sex-matched controls (age: ANOVA, *P* = 0.449; sex: Chi-square test, *P* = 0.550) (***Fig. 1A***). Demographic and clinical characteristics of the participants are summarized in ***Table 1***. Patients were selected based on predefined inclusion and exclusion criteria to ensure a well-defined and representative patient population. Inclusion criteria were: (1) laboratory-confirmed SFTS diagnosis by DBV nucleic acid testing; (2) sample collection during the acute phase of illness (4–14 days after symptom onset); (3) availability of complete clinical history and laboratory records; (4) classification as severe cases if they met at least one of the following criteria^[3]^: severe neuropsychiatric symptoms; platelet count < 50 × 10^9^/L; aspartate aminotransferase, lactate dehydrogenase, and creatine kinase levels > 5× upper limit of normal; significant hemorrhage or secondary infections; multiple organ dysfunction; or fatal outcomes. Among severe cases, those not receiving glucocorticoids were assigned to the CE group, and those receiving glucocorticoids were assigned to the PT group; (5) ordinary cases were defined as confirmed SFTS patients who did not meet any severe criterion. Exclusion criteria included: (1) concurrent tick-borne diseases or other viral hemorrhagic fevers; (2) hematologic or connective tissue disorders affecting platelet or white blood cell counts; (3) major systemic illness or chronic immunosuppressive therapy; (4) malignancy; (5) human immunodeficiency virus (HIV) infection; (6) history of blood transfusion; (7) incomplete medical records. This study was approved by the Ethics Committee of the First Affiliated Hospital of Nanjing Medical University (Approval No. 2022-SR-366).

**Table 1:** Clinical characteristics of the enrolled HC subjects and SFTS patients in the scRNA-seq analysis

Participants	Groups	Sex	Age (years)	Days from onset to sampling	Virus load (copies/mL)	Count of platelets (10^9^/L)	AST (URL)	LDH (URL)	CK (URL)	Neurological symptoms	Glucocorticoid therapy	Prognosis
Matrix1	HC	M	55.00	–	0	185.00	< 1.00	< 1.00	< 1.00	Negative	–	Health
Matrix2	HC	M	52.00	–	0	223.00	< 1.00	< 1.00	< 1.00	Negative	–	Health
Matrix3	HC	M	52.00	–	0	169.00	< 1.00	< 1.00	< 1.00	Negative	–	Health
Matrix4	OR	M	76.00	5.00	7.08 × 10^5^	55.00	2.86	2.51	3.42	Negative	No	Improvement
Matrix5	OR	F	66.00	6.00	8.10 × 10^4^	63.00	< 1.00	1.22	< 1.00	Negative	No	Improvement
Matrix6	OR	F	53.00	5.00	1.51 × 10^5^	59.00	1.04	1.32	1.35	Negative	No	Improvement
Matrix7	OR	M	78.00	7.00	9.81 × 10^6^	79.00	4.8	3.06	1.58	Negative	No	Improvement
Matrix8	OR	F	58.00	9.00	7.82 × 10^5^	104.00	2.38	1.38	< 1.00	Negative	No	Improvement
Matrix9	CE	F	49.00	9.00	1.40 × 10^7^	30.00	16.58	19.77	3.73	Drowsiness	No	Improvement
Matrix10	CE	M	61.00	6.00	4.01 × 10^7^	26.00	9.39	4.05	3.02	Drowsiness	No	Improvement
Matrix11	CE	M	67.00	7.00	2.06 × 10^6^	78.00	7.38	3.11	2.19	Tremor	No	Improvement
Matrix12	CE	M	45.00	8.00	1.02 × 10^6^	75.00	1.64	1.77	1.18	Convulsions	No	Improvement
Matrix13	CE	F	76.00	10.00	7.04 × 10^7^	34.00	8.65	14.24	2.70	Drowsiness	No	Improvement
Matrix14	PT	M	68.00	10.00	6.08 × 10^7^	35.00	41.92	11.19	4.60	Drowsiness	Yes	Improvement
Matrix15	PT	M	47.00	7.00	5.31 × 10^6^	26.00	14.93	6.1	30.82	Drosiness	Yes	Improvement
Matrix16	PT	F	67.00	5.00	3.07 × 10^7^	49.00	1.82	3.31	2.83	Drowsiness	Yes	Improvement
Abbreviations: AST, aspartate aminotransferase; CE, severe cases without glucocorticoid therapy; CK, creatine kinase; F, female; HC, healthy controls; LDH, lactate dehydrogenase; M, male; OR, ordinary SFTS cases; PT, severe cases receiving glucocorticoid therapy; SFTS, severe fever with thrombocytopenia syndrome; URL, upper reference limit.												

### Single-cell suspension preparation of PBMCs

PBMCs were isolated by density gradient centrifugation using Ficoll-Paque Plus medium and washed with Ca^2+^/Mg^2+^-free phosphate-buffered saline (PBS). Red blood cells were lysed by adding 2 mL of red blood cell lysis buffer and incubating at 25 ℃ for 10 min. The mixture was then centrifuged at 500 *g* for 5 min and the pellet was resuspended in PBS. Following this, the cell suspension was centrifuged at 400 *g* for 5 min at 4 ℃, and the supernatant was discarded. After removing red blood cells, PBMCs were purified by centrifugation at 400 *g* for 10 min at 4 ℃. The supernatant was discarded, and the PBMCs were resuspended in PBS to obtain a single-cell suspension. Finally, samples were stained with Trypan Blue, and cell viability was assessed microscopically.

### Library preparation and scRNA-seq of PBMCs

Single-cell suspensions (2 × 10^5^ cells/mL) in PBS were loaded onto a microwell chip using the Singleron scRNA-seq platform (Singleron Biotechnologies, Nanjing, Jiangsu, China)^[14]^. Barcoding beads were collected from the chip, and the mRNA captured on these beads underwent reverse transcription to generate cDNA, followed by PCR amplification. The amplified cDNA was then fragmented and ligated to sequencing adapters. The scRNA-seq libraries were prepared according to the manufacturer's protocol for the Single Cell RNA Library Kits. Individual libraries were diluted to 4 nmol/L, pooled, and sequenced on an Illumina NovaSeq 6000 platform (Illumina, San Diego, CA, USA) using 150-bp paired-end reads.

### Primary processing and quality control of scRNA-seq data

Raw sequencing reads were processed to generate gene expression matrices using the CeleScope pipeline (v1.9.0; https://github.com/singleron-RD/CeleScope). Briefly, low-quality reads were removed using CeleScope, followed by trimming of poly-A tails and adapter sequences with Cutadapt (v1.17). Cell barcodes and unique molecular identifiers (UMIs) were then extracted. Reads were aligned to the reference genome GRCh38 (Ensembl annotation version 92) using STAR (v2.6.1a). Gene-level UMI counts per cell were quantified with featureCounts (v2.0.1) and used to construct the gene expression matrix for subsequent analysis. Quality control filtering was applied to exclude cells with fewer than 200 detected genes, those in the top 2% of total gene counts, the top 2% of total UMI counts, or those with mitochondrial gene content exceeding 20%.

### Bioinformatics and statistical analysis

Single-sample gene set enrichment analysis (ssGSEA) was used to assess pathway enrichment across different groups. High-dimensional weighted correlation network analysis (hdWGCNA) was used to analyze the functional states of each cell subset^[15]^. Cell–cell communication and associated intracellular signaling pathways were inferred with CellCall, based on ligand–receptor (L–R) interactions between cell types or subtypes^[16]^. Differences in cell proportions between the two groups were compared using unpaired, two-tailed Wilcoxon rank-sum tests. Comparisons of gene expression or gene signatures between two groups of cells were performed using an unpaired, two-tailed Student's *t*-test. Comparisons of cell distribution among groups were performed using ANOVA and two-tailed *t*-tests. All of the above statistical analyses were performed using the R programming language. Correlation analysis was performed using Spearman correlation analysis with SPSS 27.0 (IBM, Armonk, NY, USA). Statistical significance was set at *P* < 0.05.

## Results

### Decline of lymphoid cell proportion within PBMCs during the SFTS acute phase

Single-cell RNA sequencing using the Singleron platform was performed on 16 PBMC samples from participants in the HC, OR, CE, and PT groups (***Fig. 1B***). A total of 214726 high-quality cells were obtained (7500–27777 cells per sample), with detection of 33406 gene features. With unbiased clustering, uniform manifold approximation and projection (UMAP) visualization and canonical gene markers, 15 clusters of immune-related cells were identified (***Fig. 1C***–***1E*** and ***Supplementary Fig. 1***). The canonical gene markers used to identify the 15 immune-related cell clusters in PBMCs are depicted in ***Supplementary Table 1***. PBMCs were isolated using Ficoll density gradient centrifugation. After centrifugation, the erythrocyte layer contained the high-density neutrophils, and the PBMC layer contained the low-density neutrophils. Consequently, the PBMC-derived immune cell clusters included the low-density neutrophil subset.

We observed that the proportion of myeloid cells gradually increased, while that of lymphoid cells gradually decreased, from the HC group to the OR, CE, and PT groups (***Fig. 1F*** and ***1G***). The lymphocyte-to-monocyte ratio (L/M ratio) from the complete blood count (CBC) data was positively correlated with that derived from scRNA-seq (***Fig. 1H***, ***Supplementary Table 2***, and ***Supplementary Fig. 2***), suggesting that lymphocyte depletion is associated with SFTS severity. Glucocorticoid treatment may further exacerbate this lymphopenia.

### Subsets in lymphoid cells

**Figure 2:**
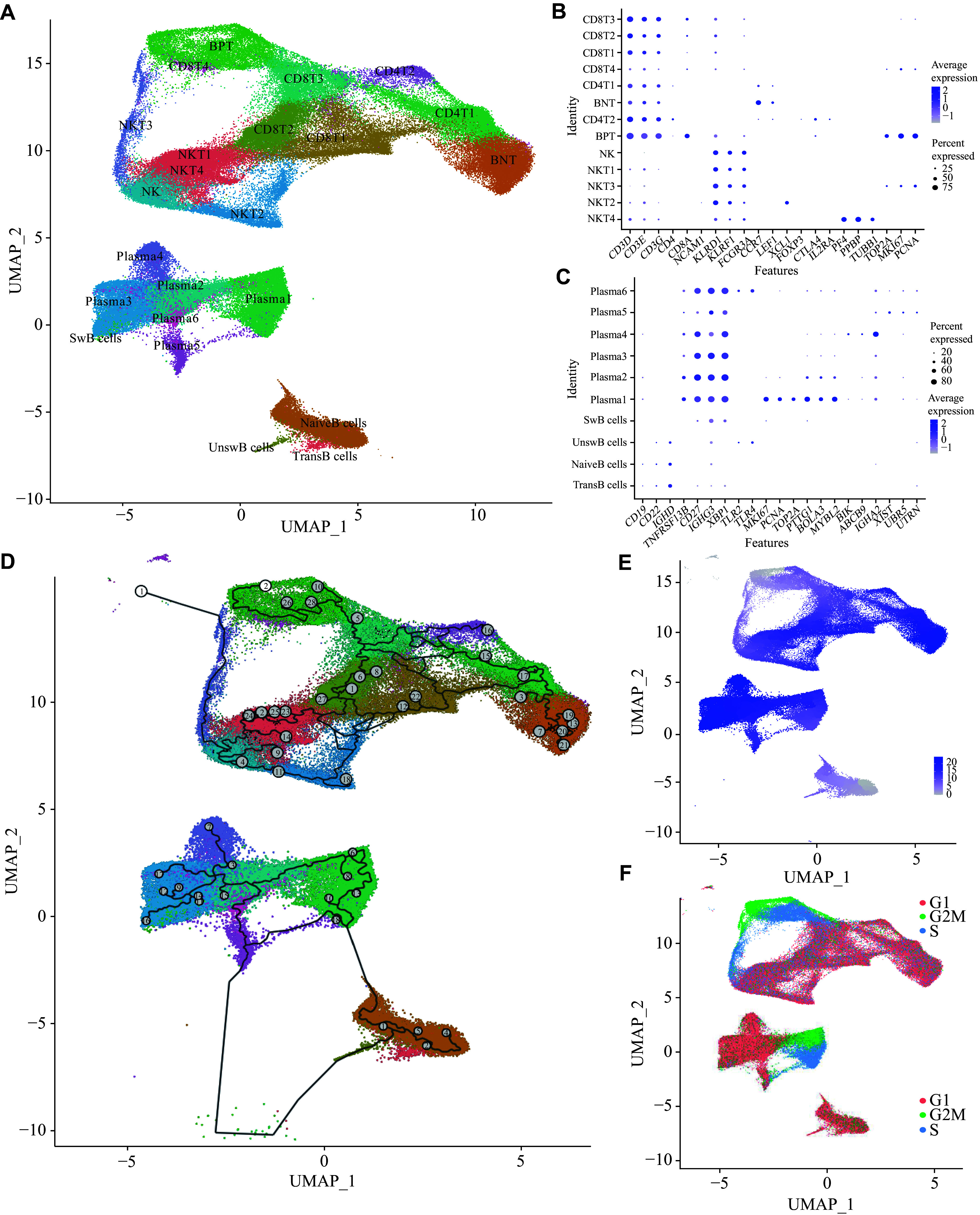
Composition and trajectory analysis of lymphoid cells. A: Uniform manifold approximation and projection (UMAP) analysis identified 23 major clusters of lymphoid cells. Data represent 120886 cells from 16 biologically independent samples. B: Dot plot depicting expression of canonical gene markers used to identify T lymphocyte clusters in peripheral blood mononuclear cells (PBMCs). C: Dot plot depicting expression of canonical gene markers used to identify B lymphocyte clusters in PBMCs. D: Trajectory simulation plot of lymphoid cells. Each dot in the graph represents a cell, and numbers in the circle represent the nodes corresponding to different cell states in the trajectory analysis. E: Trajectory simulation plot of lymphoid cells. Different colors represent different cell clusters, and the color from light to dark indicates the evolutionary direction. F: Cell cycle plot of lymphoid cells. Different colors indicate different cell cycles.

To further clarify the composition of lymphoid cells, particularly T cells, B cells, and natural killer (NK) cells, in PBMCs from SFTS patients during the acute stage, 120886 high-quality lymphoid cells (77830 T cells, 37722 B cells, and 5334 NK cells) were subdivided into 23 subclusters according to differences in gene expression (33406 gene features; ***Fig. 2A***–***2C***). The canonical gene markers used for annotating these lymphoid subsets are depicted in ***Supplementary Table 3***.

### Potential differentiation trajectories of lymphoid subsets

Trajectory analysis identified the natural killer T cells (NKT3) and bipositive T cells (BPT) subsets as the least differentiated populations for T cells and NKT cells in peripheral blood, respectively (***Fig. 2D*** and ***2E***). These subsets exhibited elevated expression of the proliferation markers *TOP2A*, *MKI67*, and *PCNA* (***Fig. 2B***) and were enriched in the G2/M or S phases of cell-cycle (***Fig. 2F***), consistent with a primitive T- or NKT-cell phenotype. In contrast, the terminal subset of T cells, binegative T cells (BNT), displayed a *CD3*^+^*CD4*^−^*CD8*^−^ phenotype lacking expression of exhaustion markers (*PD-1* and *CTLA-4*), Treg signatures (*FOXP3*), and NKT markers (*NCAM1*, *KLRD1*, *KLRF1*, and *FCGR3A*), but exhibited expression of *CCR7* and *LEF1* with G1-phase predominance^[17]^ (***Fig. 2F***), indicating that BNT cells represent a unique, terminally differentiated, and phenotypically mature T-cell subset in the peripheral blood of SFTS patients. Moreover, the BNT subset exhibited potential for lymphoid homing and activation-induced differentiation, suggesting possible roles in immune surveillance and immune activation, and warrants further functional characterization.

Trajectory analysis of B cells identified initiation nodes in transitional B (TransB) and naive B (NaiveB) subsets (***Fig. 2D***), which showed the least mature phenotypes (***Fig. 2E***) with predominantly G1-phase cells and a small proportion of S/G2M cells (***Fig. 2F***) in peripheral blood, consistent with the unstimulated naive mature B-cell state. The intermediate Plasma1 subset (***Fig. 2D***) exhibited a characteristic gene expression profile, *CD27*^+^*IGHD*^−^*IGHM*^−^*IGHG3*^+^, along with proliferation markers *MKI67*^+^*PCNA*^+^*TOP2A*^+^, aligning with reported plasmablast expansion in SFTS^[12]^. Building upon established evidence that splenic marginal zone B cells (IgM^+^) directly differentiate into plasma cells to mediate early anti-pathogen responses, our trajectory analysis identified a special unswitched B (UnswB)-to-Plasma5 developmental path, despite the absence of nodal points^[18]^ (***Fig. 2D***). This finding suggests that DBV infection may induce plasmacytic differentiation of peripheral blood B cells irrespective of their switched or unswitched status.

### Lymphoid subset redistribution by SFTS severity and glucocorticoid therapy

**Figure 3:**
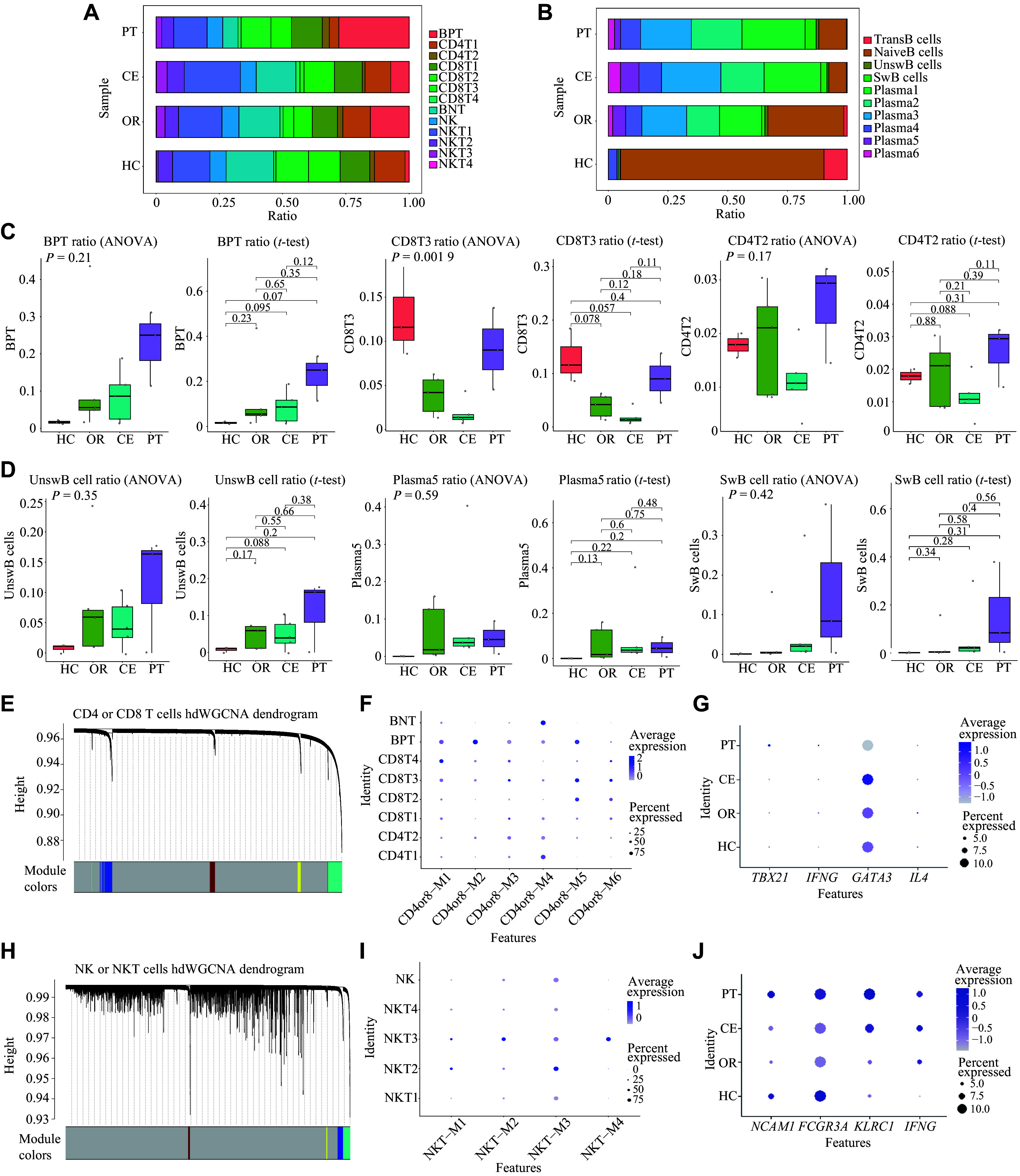
Analysis of subset proportion and gene modules in lymphoid cells. A and B: Bar charts depict the composition of T lymphocytes (A) and B lymphocytes (B) of patients with SFTS across HC, OR, CE, and PT groups. C and D: Box plots showing the ratio of T lymphocytes (C) and B lymphocytes (D) in PBMCs among patients in the HC, OR, CE, and PT groups. The box represents median ± interquartile range (IQR). Statistical analysis was performed using ANOVA and two-tailed Student's *t*-tests. E: High-dimensional weighted correlation network analysis (hdWGCNA) identified a total of six statistically significant coexpressed gene modules by targeting CD4^+^ T cells and CD8^+^ T cells. F: Dot plot showing the expression of T lymphocyte subsets in each co-expressed gene module. G: Dot plot depicting the expression of canonical gene markers used to identify the CD4T1 cell subset in PBMCs. H: hdWGCNA identified a total of four statistically significant coexpressed gene modules by targeting NK cells and NKT cells. I: Dot plot showing the expression of NK cells and NKT cells in each coexpressed gene module. J: Dot plot depicting the expression of canonical gene markers used to identify NK cell subsets in PBMCs. Abbreviations: CE, severe cases without glucocorticoid therapy; NK, natural killer cells; NKT, natural killer T cells; HC, healthy controls; OR, ordinary cases; PT, severe cases receiving glucocorticoid therapy.

Although no significant statistical differences were observed, the following trends were summarized. The proportion of NK cells, CD4^+^ T cells, and CD8+ T cells was reduced in the CE group compared with the OR group (***Fig. 3A***, ***3C***, and ***Supplementary Fig. 3***). In the PT group, the proportion of NK, CD4T1, and CD8T4 subsets increased, with the proportion of CD8T4 subset increasing more markedly, while the proportion of CD4T2 subset changed little (***Fig. 3A***, ***3C***, and ***Supplementary Fig. 3***). The proportion of BNT decreased, but that of BPT increased in the PT group, suggesting that glucocorticoids may delay T cell senescence and promote naive cell proliferation (***Fig. 3A***, ***3C***, and ***Supplementary Fig. 3***). All NKT subsets declined in the PT group compared with the CE group, especially the NKT4 subset, which revealed that glucocorticoids inhibited NKT-cell proliferation and their binding with platelets, thereby reducing cytokine production and inhibiting inflammation (***Supplementary Fig. 3***). Furthermore, the proportion of plasma cells and switched B (SwB) cells gradually increased from the HC group to the CE group, whereas TransB cells and NaiveB cells gradually decreased (***Fig. 3B***, ***3D***, and ***Supplementary Fig. 4***). The proportion of UnswB cells increased in the OR and CE groups compared with the HC group, but decreased in the CE group compared with the OR group, and increased again in the PT group compared with the CE group (***Fig. 3B***, ***3D***, and ***Supplementary Fig. 4***). This result demonstrates that UnswB cell proliferation in the CE group could not keep pace with their conversion into plasma cells, but glucocorticoids may induce the proliferation of UnswB cells, which could improve the conversion to effector plasma cells^[18]^.

### Distinct gene modules and functional programs in lymphoid subsets during SFTS

**Figure 4:**
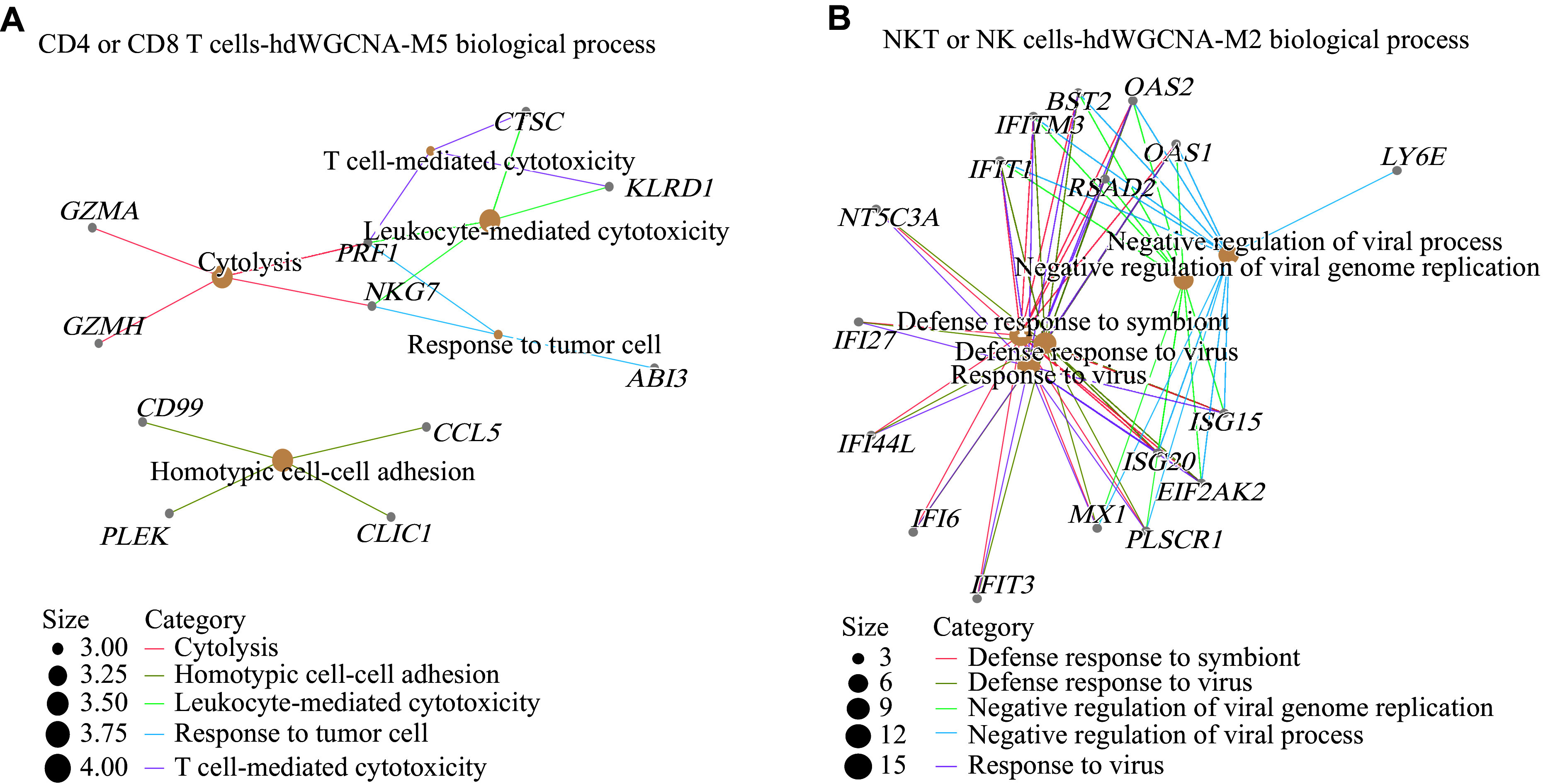
Gene Ontology (GO) analysis of the top 20 genes in gene modules from high-dimensional weighted correlation network analysis (hdWGCNA). A: GO enrichment analysis of the CD4^+^ or CD8^+^ T cell-associated module hdWGCNA-M5 (CD4 or 8-M5), showing enrichment of the term leukocyte-mediated cytotoxicity. B: GO enrichment analysis of the NKT or NK cell-associated module hdWGCNA-M2 (NKT-M2), showing enrichment of the term antiviral response.

To further clarify the function of each subset, we performed hdWGCNA on lymphoid cells. Focusing on CD4^+^ and CD8^+^ T cells, a total of six statistically significant co-expressed gene modules were identified (***Fig. 3E***, ***3F***, and ***Supplementary Fig. 5***). Similarly, four statistically significant co-expressed gene modules were identified from NK and NKT cells (***Fig. 3H***, ***3I***, and ***Supplementary Fig. 6***). Gene Ontology (GO) analysis was carried out with the top 20 genes to annotate each module function (***Fig. 4*** and ***Supplementary Fig. 7***).

Among CD4^+^ T cells with antigen-presenting capacity, the CD4T1 subset showed active cytoplasmic translation and cytokine production (***Fig. 3F*** and ***Supplementary Fig. 7D***). In the CE group, the CD4T1 subset exhibited high expression of the transcription factor *GATA3*, playing a role in humoral immunity as a T helper 2 (Th2)^[19]^ (***Fig. 3G***). The CD4T2 subset displayed regulatory T cell features, marked by specific expression of *FOXP3* and *CTLA4*, and was associated with cytokine production and antiviral responses^[20]^ (***Figs. 2B***, ***3F***, ***Supplementary Fig. 7C***, and ***7D***). GO analysis revealed that CD8T2 and CD8T3 subsets functioned as major effector T cells, exhibiting strong leukocyte-mediated cytotoxicity and antigen presentation (***Figs. 3F***, ***4A***, and ***Supplementary Fig. 7E***). Both CD8T1 and CD8T3 subsets exhibited strong antiviral responses (***Fig. 3F*** and ***Supplementary Fig. 7C***). Notably, the CD8T4 subset showed high expression of erythroid genes (*HBB*, *HBA1*, and *HBA2*), suggesting potential contamination by erythroid cells; however, it also displayed strong antigen-presenting capacity (***Fig. 3F***, ***Supplementary Fig. 7A***, and ***7E***). The BPT subset likely represents proliferating cells, characterized by high expression of cell cycle markers *TOP2A*, *MKI67,* and *PCNA*^[21]^ (***Figs. 2B***, ***3F***, and ***Supplementary Fig. 7B***). In contrast, the BNT subset exhibited strong cytoplasmic translation activity, consistent with a highly differentiated state (***Fig. 3F*** and ***Supplementary Fig. 7D***). Among NKT cells, NKT2 emerged as the primary effector subset, showing strong cytoplasmic translation and leukocyte-mediated immune responses (***Fig. 3I***, ***Supplementary Fig. 7F***, and ***7G***). The NKT3 subset, enriched for *TOP2A*, *MKI67,* and *PCNA*, represented cycling cells with enhanced proliferative and antiviral functions (***Figs. 2B***, ***3I***, ***4B***, and ***Supplementary Fig. 7H***). Finally, the NKT4 subset specifically expressing *PF4*, *PPBP,* and *TUBB1* may present a combination of NKT cells and platelets, which still requires further verification^[22]^ (***Fig. 2B***).

In our study, we focused on single-cell transcriptomic sequencing analysis of the NK cell subset involved in innate immunity. The results showed that, following DBV infection, NK cells primarily mediated antibody-dependent cellular cytotoxicity *via* the *FCGR3A* gene^[23]^ (***Figs. 2B***, ***3I***, ***4B***, and ***Supplementary Fig. 7G***). Concurrently, these cells exhibited low expression of the *NCAM1* gene, which is associated with the modulation of IFN-γ production, with the highest IFN-γ expression noted in the CE group^[24]^. Glucocorticoid treatment suppressed both *IFN-α* and *IFN-γ* expression. However, it upregulated *KLRC1*, which may pair with HLA-E to form immune receptors that distinguish "self" from "non-self", thereby enhancing NK cell self-tolerance^[25]^ (***Fig. 3J***).

In several B-cell subsets, cell numbers were insufficient for robust gene co-expression network analysis; therefore, we integrated canonical marker genes with trajectory inference to infer subset functions (***Fig. 2***). Plasma1, characterized by high expression of *TOP2A*, *MKI67,* and *PCNA*, represented cycling cells. Plasma2 with specific expression of *PTTG1*, *BOLA3*, and *MYBL2* represented cells in the G1 phase^[26]^. Plasma3 highly expressed *IGHG3*, suggesting that it produces antibodies primarily against biological macromolecules such as pathogen-associated molecular patterns (PAMPs). Plasma4, marked by high levels of *BIK*, *ABCB9*, *IGHA1*, and *IGHA2*, represented an IgA-producing cell subset^[27]^. Plasma5 specifically expressed *XIST* and *UBR5*, representing virus-specific plasma cells^[28]^. Plasma6 specifically expressed *IL4R*, *UTRN*, *TLR4*, and *TLR2*, indicating a role in generating antibodies against damage-associated molecular patterns (DAMPs)^[29]^. SwB cells represented memory B cells generated following DBV infection.

### Lymphoid hyperinflammation versus glucocorticoid immunosuppression in severe SFTS

**Figure 5:**
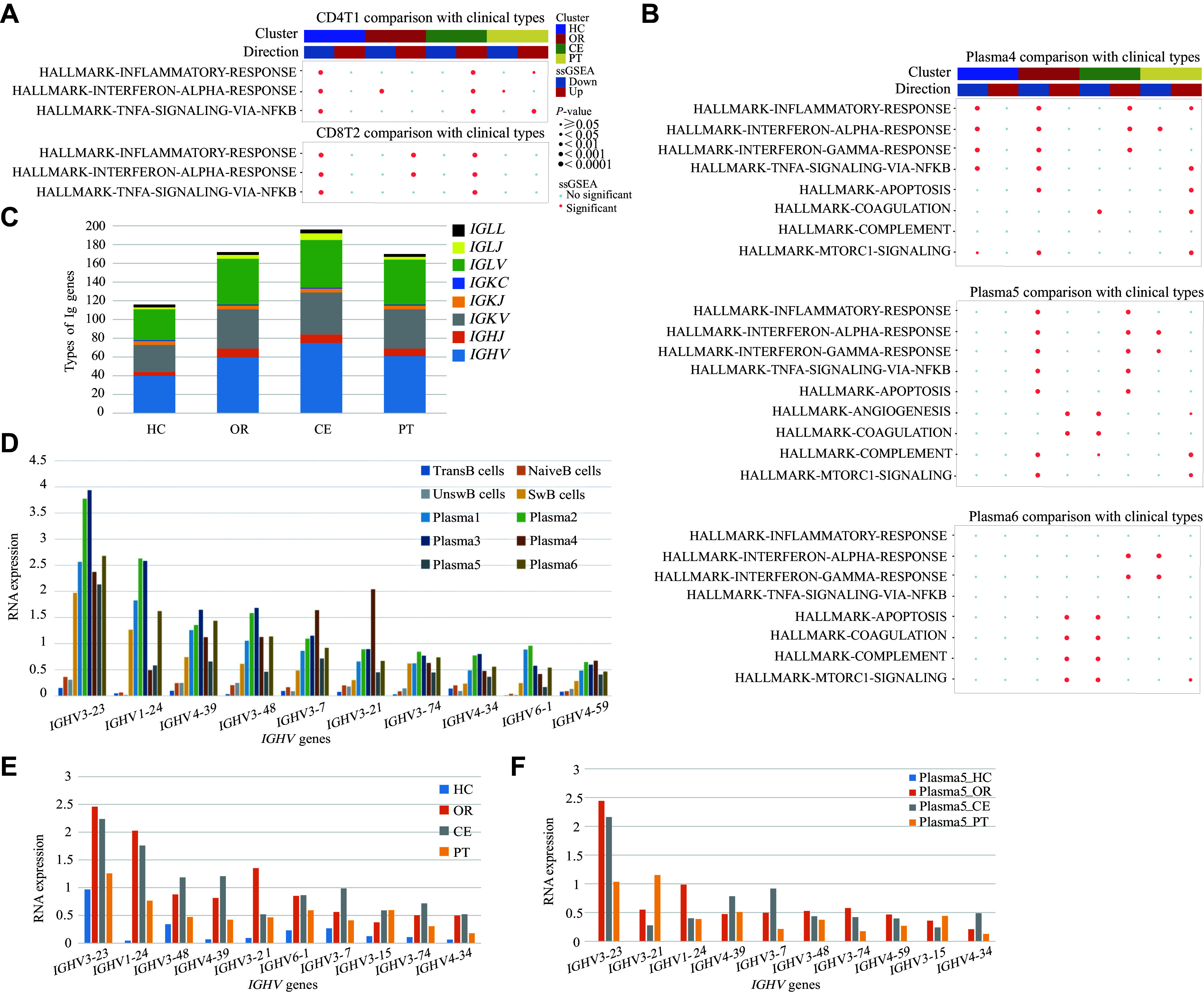
Single-sample gene set enrichment analysis (ssGSEA) of lymphoid cells and analysis of immunoglobulin (Ig) gene components. A: ssGSEA of CD4T1 and CD8T2 subsets, depicting intergroup transcriptional perturbations in canonical signaling pathways associated with inflammation. B: ssGSEA of Plasma4, Plasma5, and Plasma6 subsets, showing intergroup transcriptional perturbations in canonical signaling pathways related to inflammation, complement, coagulation, and autophagy regulation. C: Bar chart showing the diversity of Ig genotypes. D: Expression of *IGHV* genes in B lymphocyte subsets. E: Expression of *IGHV* genes among patients in the HC, OR, CE, and PT groups. F: Expression of *IGHV* genes in Plasma5 cells among patients in the HC, OR, CE, and PT groups. Abbreviations: CE, severe cases without glucocorticoid therapy; HC, healthy controls; OR, ordinary cases; PT, severe cases receiving glucocorticoid therapy.

The ssGSEA analysis revealed that IFN-α and inflammation-related gene signatures were active across most T cell subsets, with the notable exceptions of CD4T2, CD8T3, and CD8T4. These signatures were further elevated in the CE group compared with the HC and OR groups but were suppressed in the PT group compared with the CE group (***Fig. 5A*** and ***Supplementary Fig. 8***). Notably, this suppression was absent in CD8T3 and CD8T4 cells in the PT group. Plasma cells exhibited a similar IFN-α, IFN-γ, and inflammatory profile to that of T cells. In addition, genes involved in complement activation, coagulation, and apoptosis were significantly downregulated in plasma cells in the CE group compared with the HC and OR groups, but were upregulated in the PT group (***Fig. 5B*** and ***Supplementary Fig. 9***).

The results showed that interferon and inflammation responses were upregulated in severe SFTS patients, which was beneficial for the antiviral response. Glucocorticoid treatment reversed this pro-inflammatory state and effectively suppressed inflammation. It also enhanced complement activation, coagulation, apoptosis, and mTORC1 signaling in lymphocytes, promoting the clearance of cells infected by DBV^[30]^. However, elevated mTORC1 signaling may inhibit cellular autophagy and hinder virus clearance^[31]^.

### Biphasic plasma cell responses: antiviral antibody production versus glucocorticoid suppression

Following DBV infection, both the diversity and expression of immunoglobulin (Ig) genes were significantly increased (***Fig. 5C***). *IGHV* and *IGLV* gene expression followed consistent patterns across B-cell subsets (***Supplementary Fig. 10A***), with lower expression in NaiveB cells and TransB cells but higher expression in SwB cells and plasma cells, particularly in Plasma2 and Plasma3 cells (***Fig. 5D***). The expression of *IGHV* and *IGLV* genes, such as *IGHV3-23*, *IGHV1-24*, *IGLV2-14,* and *IGLV1-47*, was at basal levels in the HC group, remained consistently high in the OR and CE groups, and decreased in the PT group (***Fig. 5E*** and ***Supplementary Fig. 10B***). The expression of Ig genes showed a weaker increasing trend in Plasma5 cells than in other plasma cells. Notably, *IGHV* and *IGKV* expression in Plasma5 was detectable only in the OR, CE, and PT groups. We propose that Plasma5 cells were activated following DBV infection and subsequently produced virus-specific antibodies in the OR and CE groups. However, after glucocorticoid treatment, the levels of *IGHV* and *IGLV* expressed by Plasma5 cells were both decreased, suggesting that glucocorticoid treatment inhibits the production of virus-specific antibodies^[32]^ (***Fig. 5F*** and ***Supplementary Fig. 10C***).

### Lymphoid interactome reprogramming in SFTS progression and glucocorticoid therapy

**Figure 6:**
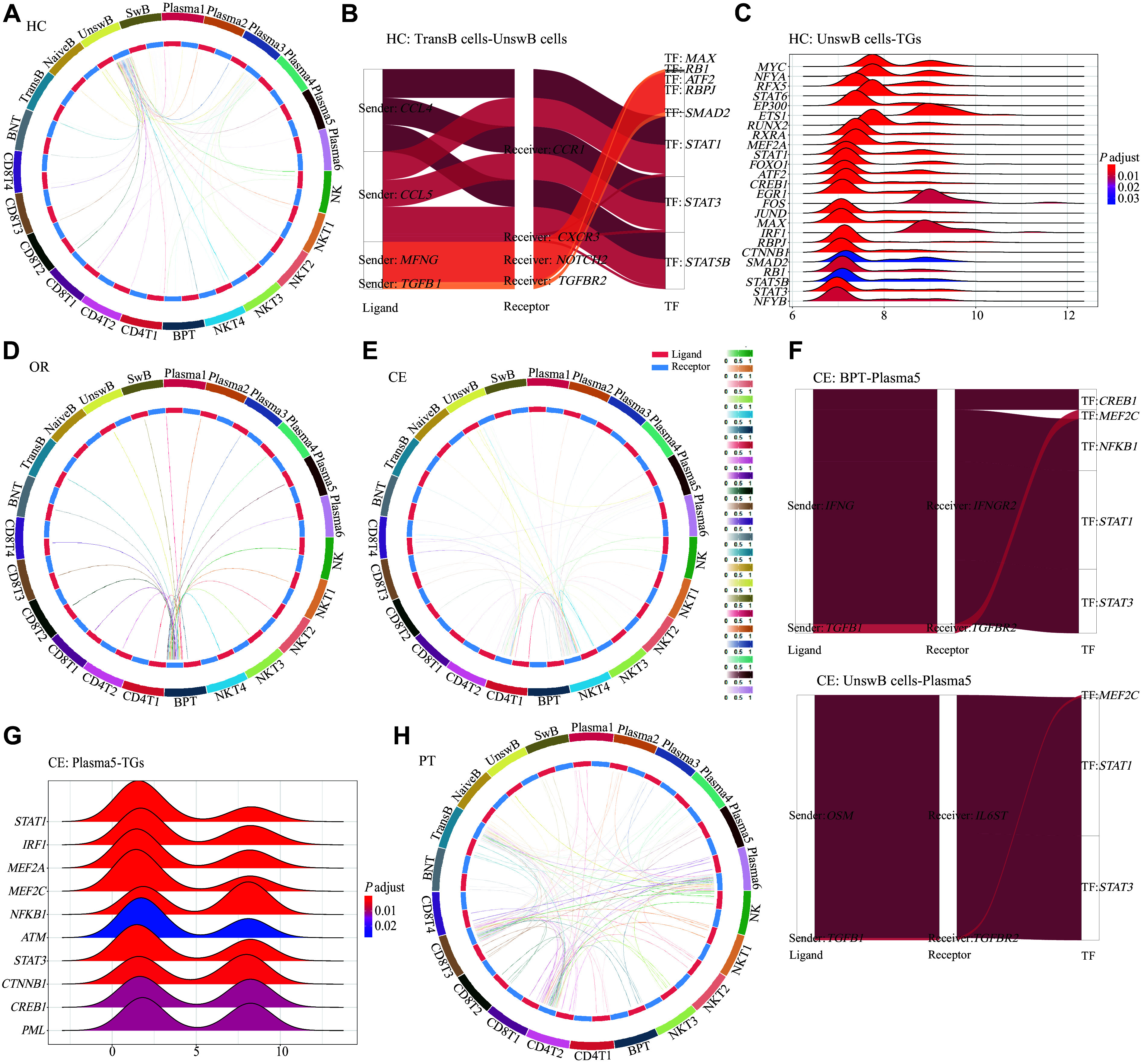
Heterogeneity of lymphoid cell interaction during the acute phase of SFTS. A: Circos plot of intercellular communication from lymphoid cells in the HC group. The inner circle shows the cell receiving area (blue) and sending area (red). B: Sankey diagram describing the ligand–receptor (L‒R) pairs and TFs of the receptor cells, unswitched B (UnswB) cells, in the HC group. C: Ridge plot showing the fold-change (FC) distribution of the downstream TGs activated by the TFs of UnswB cells in the HC group. D: Circos plot of intercellular communication from lymphoid cells in the OR group. E: Circos plot of intercellular communication from lymphoid cells in the CE group. F: Sankey diagram describing the L‒R pairs and the TFs of the receptor cells, Plasma5 cells, in the CE group. G: Ridge plot showing the FC distribution of the downstream TGs activated by the TFs of Plasma5 cells in the CE group. H: Circos plot of intercellular communication from lymphoid cells in the PT group. Abbreviations: BPT, bipositive T cells; CE, severe cases without glucocorticoid therapy; HC, healthy controls; OR, ordinary cases; PT, severe cases receiving glucocorticoid therapy; TF, transcription factor; TGs, target genes (regulated by TF).

Seven recipient cell subsets were predominantly enriched in the HC group, including UnswB, Plasma3, BPT, Plasma1, Plasma2, Plasma4, and SwB cells (***Fig. 6A***). The transcription factors (TFs) of UnswB cells, such as *RBPJ*, *SMAD2*, *STAT1*, *STAT3*, *STAT5B*, and *STAT6*, were activated by the cellular senescence and MAPK pathways, subsequently driving the expression of downstream target genes (TGs) such as *AP-1*, *NFYB*, *IRF4*, *MYC*, *SMAD2*, *ETS1*, and *IRF1*^[33]^ (***Fig. 6B*** and ***6C***). Among these, *NFYB*, *MYC*, and *SMAD2* are mainly related to cell proliferation^[34]^; *STAT3* and *STAT5B* are associated with cell development and differentiation; *IRF4* and *ETS1* promote B cell maturation and plasma cell differentiation^[35]^; and *IRF1* initiates interferon-mediated immune responses critical for antiviral defense^[31]^. Plasma3 cells had similar effects, suggesting that differentiation and development from UnswB to Plasma3 cells represent the fundamental mechanism of humoral immunity in healthy people (***Supplementary Fig. 11B*** and ***11C***).

BPT cells were predominantly enriched as recipient cells in the OR group (***Fig. 6D***). Signals received by receptors were further transmitted to the TFs, such as *FOXM1* and *MYBL2,* through the cellular senescence pathway^[36]^ (***Supplementary Fig. 11F***). Downstream TGs, such as *GATA3* and *BRCA1*, were then activated to promote the differentiation of BPT cells into effector T cells^[37]^ (***Supplementary Fig. 11G***).

Eight recipient cell subsets were predominantly enriched in the CE group, including NKT4, CD4T1, Plasma5, CD8T4, CD4T2, UnswB, SwB, and BPT cells (***Fig. 6E***). In addition to TGs linked to cell proliferation and differentiation (*NFYB*, *CREB1*, *STAT*, *FOXM1*, *ETS1,* and *GATA3*), inflammation-related TGs, such as *AP-1*, *RELA*, *NFKB1*, *HIF1A,* and *FOXO1*, were also activated^[38]^ (***Fig. 6F*** and ***6G***). The enhanced signal-receiving capacity of CD4T1, CD8T4, and Plasma5 cells suggests the activation of acquired immunity (***Supplementary Fig. 12B*** and ***12C***). The enhanced signal-receiving capacity of NKT4 cells, a conjugate of NKTs and platelets, suggests enhanced cytokine secretion and potential involvement of platelets (***Supplementary Fig. 12D***).

Eight recipient cell subsets were mainly enriched in the PT group, including CD4T2, Plasma6, CD8T3, TransB, Plasma4, CD8T4, Plasma1, and BPT cells (***Fig. 6H***). The activation of TGs related to cell proliferation, differentiation, and inflammation was similar to that in the CE group. Enhanced signal-receiving capacity in CD4T2 cells was linked to suppression of the activation and proliferation of autoreactive T cells (***Supplementary Fig. 13B***). In contrast, NKT4 cells showed absent signal reception, indicating that the binding of NKT to platelets was inhibited. The enhanced receiving signal of CD8T3 cells indicated that the cytotoxic effects of humoral immunity were strengthened (***Supplementary Fig. 13C***). The enhanced receiving signals of TransB, Plasma6, and Plasma4 cells suggested that humoral immunity mainly targeted DAMPs and stimulated IgA secretion^[18]^ (***Supplementary Fig. 13D***).

## Discussion

In this study, we presented an integrated single-cell landscape of lymphoid cells in peripheral immune responses and revealed marked changes in the cell composition and cell-cell interactions during DBV infection. Notably, distinct immune characteristics were identified in ordinary SFTS patients and severe SFTS patients receiving glucocorticoid treatment.

The CE group exhibited a stronger inflammatory response and acquired immune response compared with the OR group. The IFN-α, IFN-γ, and inflammatory responses of lymphoid cells were upregulated or remained steady in the OR group compared with the HC group. The increase of IFN-α expression levels may exacerbate inflammation and aggravate illness in SFTS patients, which has also been reported in severe COVID-19 patients^[39]^. Only BPT cells were enriched as recipient cells in the interaction among lymphoid subsets, indicating that acquired immunity was not fully activated. However, the IFN-α, IFN-γ, and inflammatory responses of lymphoid cells in the CE group were further enhanced compared with those in the OR group. Recipient cells enriched in the CE group, such as helper T cells (CD4T1) and Plasma5 cells, suggested the activation of acquired immunity. Plasma5 cells may be the main effector plasma cells that produce virus-specific antibodies. However, for CD8^+^ T cells, we only enriched CD8T4 cells (cycling cells) as recipient cells with enhanced proliferative activity in the analysis of lymphocytic cell-cell interactions, indicating that cellular immunity was not fully activated.

Glucocorticoid treatment can inhibit inflammatory and interferon responses while strengthening cellular immunity. IFN-α expression was found to be positively correlated with death-associated cytokines, such as CXCL10 and IL-6^[12]^. The inflammatory and interferon responses of lymphoid cells were decreased in the PT group compared with the CE group, which may be achieved by regulatory T cells (CD4T2) observed in the cellular interaction analysis. CD4T2 cells acted on CD8T3 cells, the main effector cells, to enhance cellular immunity. The proportion of CD8T cells exhibited a decreasing trend after DBV infection but showed an increase after glucocorticoid treatment. Cellular interaction analysis revealed that the NKT4 cell subset could activate the CD8T4 cells, with NKT4 potentially representing a conjugate of NKT cells and platelets. However, previous analyses suggested that the function of the CD8T4 cell subset involved gas transport, likely stemming from erythrocyte contamination. Therefore, the signaling pathway mediated by the CD8T4 subset was excluded from further analysis. Humoral immunity, primarily mediated by B cells, was prominently activated after DBV infection^[12,40]^. Conversely, cellular immunity showed significant activation following glucocorticoid treatment, with cytotoxic effects, antigen presentation reactions, and antiviral responses mediated by CD8T3 cell subset, contributing to the restoration of immune balance. The full activation of complement, coagulation, and apoptosis responses in the PT group not only strengthened the acquired immunity of the host but also exerted cytotoxic effects and coagulation, which may be favorable for the recovery of severe patients.

Glucocorticoids inhibited the expression of Ig genes, leading to a decrease in the production of virus-specific antibodies. The basal expression of Ig genes was observed across all B cell subsets, with a weaker trend noted in Plasma5 cells compared with other plasma cell subsets. Elevated expression levels of IGHV and IGLV were found in plasma cells within the OR and CE groups after DBV infection^[18,32]^. CD4T1 cells, known as Th2 cells, demonstrated the capability to stimulate and facilitate the differentiation of B cells into plasma cells through the secretion of cytokines such as IL-4, leading to the generation of DBV-specific antibodies. Our analysis of cellular interactions revealed that Plasma5 cells were predominantly activated in the CE group after DBV infection, producing antibodies primarily targeting PAMPs. Notably, the expression of diverse antibodies in Plasma5 cells exhibited a consistent increase in the OR and CE groups but declined in the PT group. Conversely, regulatory T cells CD4T2, alongside the Plasma6 and Plasma4 cell subsets, were activated in the PT group and assumed regulatory functions. Plasma6 cells were mainly activated to produce antibodies against DAMPs, while Plasma4 cells were capable of secreting IgA antibodies^[18]^. In summary, glucocorticoid treatment was found to inhibit the production of virus-specific antibodies and humoral immunity during the acute phase of SFTS. Therefore, prolonged use of high-dose glucocorticoids as anti-inflammatory therapy is not recommended.

While our single-cell transcriptomic analysis first delineated the molecular characteristics of peripheral blood lymphocytes in acute-phase SFTS patients, with key observations supported by authoritative studies, these findings require further multi-omics and functional validation (*e.g.*, protein assays and mechanistic studies) because of the inherent limitations of transcriptional data.

## Additional information

The online version contains supplementary material available at http://www.jbr-pub.org.cn/article/doi/10.7555/JBR.39.20250250?pageType=en.
