# Supplementary material for: Heterogeneity of lymphoid cells in PBMCs in the acute phase of SFTS: Single-cell transcriptome profiling
Source: J Biomed Res. 2026 Mar 19;40(2):196–209. doi: 10.7555/JBR.39.20250250 (PMC13044404; doi:10.7555/JBR.39.20250250)
Supplement: Supplementary file 1 — The online version contains supplementary material available at http://www.jbr-pub.org.cn/article/doi/10.7555/JBR.39.20250250?pageType=en. [file jbr-40-2-196-S1.pdf]

Available online at www.jbr-pub.org.cn

Open Access at PubMed Central

Journal of Biomedical Research, 2026 40(2): 1–4

Supplementary Data

JBR

Heterogeneity of lymphoid cells in PBMCs in the acute phase of SFTS: Single-cell transcriptome profiling

Jiaying Zhao[1,△], Ruowei Xu[2,3,△], Tingting Zhou[2], Ke Jin[1], Jin Zhu[2], Jinhai Zhang[2], Yifang Han[2], Xinjian Liu[4], Dafeng Lu[2,5], Chunfang Wang[2,5], Jiaojiao Qian[2,5], Chunhui Wang[2,3,5,✉], Jun Li[1,✉]

[1]Department of Infectious Diseases, the First Affiliated Hospital of Nanjing Medical University, Jiangsu Province Hospital, Nanjing, Jiangsu 210029, China;
[2]Epidemiological Department, Huadong Medical Institute of Biotechniques, Nanjing, Jiangsu 210002, China;
[3]School of Life Sciences, Nanjing Normal University, Nanjing, Jiangsu 210046, China;
[4]School of Basic Medical Sciences, Nanjing Medical University, Nanjing, Jiangsu 211166, China;
[5]School of Public Health, Nanjing Medical University, Nanjing, Jiangsu 211166, China.

Supplementary Table 1 **Canonical gene markers used to identify 15 immune-related cell clusters in peripheral blood mononuclear cells**

Cell clusters	Canonical gene markers
Neutrophils	CSF3R$^+$, FCGR3B$^+$, CXCR2$^+$, HLA-DRA$^-$, HLA-DRB1$^-$
Basophils	GATA2$^+$, CLC$^+$, MS4A2$^+$, CXCR4$^+$
Monocytes	ITGAM$^+$, CD14$^+$, HLA-DRA$^+$, HLA-DRB1$^+$
Macrophages	ITGAM$^+$, CD68$^+$, CD11C$^-$
Conventional dendritic cells (cDCs)	CD1C$^+$, CLEC10A$^+$
Plasmacytoid dendritic cells (pDCs)	ITGAM$^+$, ILR3A$^+$
Bipositive T cells (BPT)	CD3D$^+$, CD3E$^+$, CD3G$^+$, CD4$^+$, CD8A$^+$, TOP2A$^+$, MKI67$^+$, PCNA+
CD4$^+$ T cells	CD3D$^+$, CD3E$^+$, CD4$^+$
CD8$^+$ T cells	CD3D$^+$, CD3E$^+$, CD8B$^+$
Natural killer T cells (NKT)	CD3D$^+$, CD3E$^+$, NCAM1$^+$
Natural killer cells (NK)	NCAM1$^+$, KLRD1$^+$
B cells	CD79A$^+$, CD79B$^+$
Plasma cells	TNFRSF17$^+$, SDC1$^+$
Platelets	PF4$^+$, PPBP$^+$, TUBB1$^+$
Endothelial	CD34$^+$

△These authors contributed equally to this work.

✉Corresponding authors: Chunhui Wang, Epidemiological Department, Huadong Medical Institute of Biotechniques, 293 Zhongshandong Road, Nanjing, Jiangsu 210002, China; School of Life Sciences, Nanjing Normal University, 163 Xianlin Avenue, Nanjing, Jiangsu 210046, China; School of Public Health, Nanjing Medical University, 101 Longmian Avenue, Nanjing, Jiangsu 211166, China. E-mail: 13912966353@139.com; Jun Li, Department of Infectious Diseases, the First Affiliated Hospital of Nanjing Medical University, Jiangsu Province Hospital, 300 Guangzhou Road, Nanjing, Jiangsu 210029, China. E-mail: dr-lijun@vip.sina.com.

Received: 20 June 2025; Revised: 11 July 2025; Accepted: 15 July 2025; Available online: 17 July 2025; Published date: 19 March 2026

CLC number: R511, Document code: A

The authors reported no conflict of interests.

This is an open access article under the Creative Commons Attribution (CC BY 4.0) license, which permits others to distribute, remix, adapt and build upon this work, for commercial use, provided the original work is properly cited.

© 2026 by Journal of Biomedical Research.

https://doi.org/10.7555/JBR.39.20250250

Supplementary Table 2 **Ratio of lymphocytes to monocytes in complete blood count (CBC) and single-cell RNA sequencing (scRNA-seq)**

Matix	L/M ratio in scRNA-seq (%)	L/M ratio in CBC (%)	Ratio of monocytes in CBC (%)	Ratio of lymphocytes in CBC (%)	Ratio of monocytes in scRNA-seq (%)	Ratio of lymphocytes in scRNA-seq (%)
4	2.04	5.25	1.2	6.3	26.4	53.9
5	2.19	3.71	14.5	53.8	26.1	57.2
6	5.96	7.91	3.2	25.3	12.2	72.7
7	2.85	7.46	4.8	35.8	25.5	72.7
8	2.1	2.63	6.5	17.1	21.1	44.4
9	14.54	3.46	5.6	19.4	6.3	91.6
10	3.3	4.44	3.6	16	13.4	44.2
11	2.03	3.44	4.8	16.5	24.7	50.2
12	0.77	7.15	2	14.3	44.6	34.4
13	8.84	9.4	5.2	48.9	5.7	50.4
14	1.26	1.92	6.5	12.5	19.7	24.8
15	7.57	6.85	6.7	45.9	11.5	87.1
16	0.79	2.81	8.5	23.9	20.6	16.2

Supplementary Table 3 **Canonical gene markers used to identify 23 subsets in lymphoid cells**

Subsets	Canonical gene markers
NKT1	CD3D^{+}, CD3E^{+}, CD3G^{+}, KLRD1^{+}, KLRF1^{+}, FCGR3A^{+}
NKT2	CD3D^{+}, CD3E^{+}, CD3G^{+}, NCAM1^{+}, KLRD1^{+}, KLRF1^{+}, FCGR3A^{+}, XCL1^{+}
NKT3	CD3D^{+}, CD3E^{+}, KLRD1^{+}, KLRF1^{+}, FCGR3A^{+}, TOP2A^{+}, MKI67^{+}, PCNA^{+}
NKT4	CD3D^{+}, CD3E^{+}, CD3G^{+}, KLRD1^{+}, KLRF1^{+}, FCGR3A^{+}, PF4^{+}, PPBP^{+}, TUBB1^{+}
NK	CD3E^{+}, KLRD1^{+}, KLRF1^{+}, FCGR3A^{+}
Bipositive T cells (BPT)	CD3D^{+}, CD3E^{+}, CD3G^{+}, CD4^{+}, CD8A^{+}, TOP2A^{+}, MKI67^{+}, PCNA^{+}
Binegative T cells (BNT)	CD3D^{+}, CD3E^{+}, CD3G^{+}, CCR7^{+}, LEF1^{+}
CD4T1	CD3D^{+}, CD3E^{+}, CD3G^{+}, CD4^{+}, CCR7^{+}, LEF1^{+}
CD4T2	CD3D^{+}, CD3E^{+}, CD3G^{+}, CD4^{+}, CCR7^{+}, LEF1^{+}, FOXP3^{+}, CTLA4^{+}, IL2RA^{+}
CD8T1	CD3D^{+}, CD3E^{+}, CD3G^{+}, CD8A^{+}, KLRD1^{+}
CD8T2	CD3D^{+}, CD3E^{+}, CD3G^{+}, CD8A^{+}, KLRD1^{+}, FCGR3A^{+}
CD8T3	CD3D^{+}, CD3E^{+}, CD3G^{+}, CD8A^{+}, KLRD1^{+}, FCGR3A^{+}, MKI67^{+}, PCNA^{+}
CD8T4	CD3D^{+}, CD3E^{+}, CD3G^{+}, CD8A^{+}, KLRD1^{+}, FCGR3A^{+}, TOP2A^{+}, MKI67^{+}, PCNA^{+}
Transitional B (TransB) cells	TNFRSF13B^{-}, CD27^{-}, CD22^{+}, IGHD^{+}, IGHM^{+}, IGHG3^{-}, XBP1^{+}
Naive B (NaiveB) cells	TNFRSF13B^{-}, CD27^{-} CD22^{+}, IGHD^{+}, IGHM^{+}, IGHG3^{-}
Unswitched B (UnswB) cells	TNFRSF13B^{-}, CD27^{-}, CD22^{-}, IGHD^{+}, IGHM^{+}, IGHG3^{+}, TLR4/TLR2^{+}
Switched B (SwB) cells	TNFRSF13B^{-}, CD27^{+}, CD22^{-}, IGHD^{-}, IGHM^{-}, IGHG3^{+}
Plasma1	TNFRSF13B^{+}, CD27^{+}, IGHD^{-}, IGHM^{-}, IGHG3^{++}, MKI67/PCNA/TOP2A^{+}
Plasma2	TNFRSF13B^{+}, CD27^{+}, IGHD^{-}, IGHM^{-}, IGHG3^{++}, PTTG1/BOLA3/MYBL2^{+}
Plasma3	TNFRSF13B^{+}, CD27^{+}, IGHD^{-}, IGHM^{-}, IGHG3^{++}
Plasma4	TNFRSF13B^{+}, CD27^{+}, IGHD^{-}, IGHM^{-}, IGHG3^{++}, BIK/ABCB9^{+}, IGHA1/IGHA2^{++}
Plasma5	TNFRSF13B^{+}, CD27^{+}, IGHD^{-}, IGHM^{-}, IGHG3^{++}, XIST/UBR5^{+}
Plasma6	TNFRSF13B^{+}, CD27^{+}, IGHD^{-}, IGHM^{-}, IGHG3^{++}, IL4R/UTRN^{+}, TLR4/TLR2^{+}

Supplementary Fig. 1 **Composition of lymphoid cells.** A: The t-distributed stochastic neighbor embedding (t-SNE) analysis identified 15 major clusters. B: Bar chart showing the lymphoid cell composition across 16 samples (available online).

Supplementary Fig. 2 **Scatter plot of lymphocyte-to-monocyte (L/M) ratio in single-cell RNA sequencing (scRNA-seq) and complete blood count (CBC).** The outlier from matrix9, colored in purple, was removed from further correlation analysis (available online).

Supplementary Fig. 3 **Analysis of T-cell subset proportions.** Box plot showing the ratio of T lymphocytes in peripheral blood mononuclear cells (PBMCs) among patients in the healthy controls (HC), ordinary cases (OR), severe cases without glucocorticoid therapy (CE), and severe cases receiving glucocorticoid therapy (PT) groups (available online).

Supplementary Fig. 4 **Analysis of the proportion of B-cell subsets.** A: Box plot showing the composition of B lymphocytes of 16 samples. B: Box plots showing the ratio of B lymphocytes in peripheral blood mononuclear cells (PBMCs) among patients in the healthy controls (HC), ordinary cases (OR), severe cases without glucocorticoid therapy (CE), and severe cases receiving glucocorticoid therapy (PT) groups (available online).

Supplementary Fig. 5 **High-dimensional weighted correlation network analysis (hdWGCNA) and Gene Ontology (GO) analysis of CD4^{+} T cells and CD8^{+} T cells.** A: Hub gene visualization in each module ranked by kME. B: FeaturePlot depicting the distribution of 6 modules in CD4^{+} T cells and CD8^{+} T cells by dimension reduction. C: The violin plot shows the module gene expression in T cells among healthy controls (HC), ordinary cases (OR), severe cases without glucocorticoid therapy (CE), and severe cases receiving glucocorticoid therapy (PT) groups in each module. D: Scale-free network of key genes in six modules colored differently (available online).

Supplementary Fig. 6 **High-dimensional weighted correlation network analysis (hdWGCNA) and Gene Ontology (GO) analysis of natural killer T (NKT) cells.** A: FeaturePlot depicting the distribution of four modules in NKT cells by dimension reduction. B: Hub gene visualization in each module ranked by kME. C: The violin plot shows module gene expression in NKT cells in each of the four modules. D: The violin plot shows module gene expression in NKT cells among healthy controls (HC), ordinary cases (OR), severe cases without glucocorticoid therapy (CE), and severe cases receiving glucocorticoid therapy (PT) groups in each module (available online).

Supplementary Fig. 7 **Supplementary GO analysis of the top 20 genes in gene modules from high-dimensional weighted correlation network analysis (hdWGCNA).** A: Functional enrichment analysis of module CD4 or CD8 T cells-hdWGCNA-M1 (CD4 or 8-M1), indicating a role in gas transport (the contamination of erythroid cells). B: Functional enrichment analysis of module CD4 or CD8 T cells-hdWGCNA-M2 (CD4 or 8-M2), indicating a role in cell proliferation. C: Functional enrichment analysis of module CD4 or CD8 T cells-hdWGCNA-M3 (CD4 or 8-M3), indicating a role in antiviral response. D: Functional enrichment analysis of module CD4 or CD8 T cells-hdWGCNA-M4 (CD4 or 8-M4), indicating a role in cytoplasmic translation (increased cytokines). E: Functional enrichment analysis of module CD4 or CD8 T cells-hdWGCNA-M6 (CD4 or 8-M6), indicating a role in antigen presentation. F: Functional enrichment analysis of module NKT or NK cells-hdWGCNA-M1 (NKT-m1), indicating a role in cytoplasmic translation (increased cytokines). G: Functional enrichment analysis of module NKT or NK cells-hdWGCNA-M3 (NKT-m3), indicating a role in leukocyte-mediated immune response. H: Functional enrichment analysis of module NKT or NK cells-hdWGCNA-M4 (NKT-m4), indicating a role in cell proliferation (available online).

Supplementary Fig. 8 **Single-sample gene set enrichment analysis (ssGSEA) in T and natural killer T (NKT) subsets.**

Supplementary Fig. 9 **Single-sample gene set enrichment analysis (ssGSEA) of B lymphocytes.** A: Bar plot showing the proportion and count relation between the ssGSEA result and B subsets. B: Intersections between the ssGSEA result and B subsets. C: ssGSEA in each subset of B cells (available online).

Supplementary Fig. 10 **Analysis of immunoglobulin (Ig) gene components.** A: The expression of *IGLV* genes in B lymphocyte subsets. B: The expression of *IGLV* genes among patients in healthy controls (HC), ordinary cases (OR), severe cases without glucocorticoid therapy (CE), and severe cases receiving glucocorticoid therapy (PT) groups. C: The expression of *IGKV* genes in B lymphocyte subsets. D: The expression of *IGKV* genes among patients in HC, OR, CE, and PT groups. E: The expression of *IGLV* genes in Plasma5 cells among patients in HC, OR, CE, and PT groups. F: The expression of *IGKV* genes in Plasma5 cells among patients in HC, OR, CE, and PT groups (available

online).

Supplementary Fig. 11 **Cellcall analysis of lymphoid cells in the healthy controls (HC) and ordinary cases (OR) groups.** A: Circos plot of intercellular communication among lymphoid cells in the HC group. B: Sankey diagram describing the ligand–receptor (L-R) pairs and transcription factors (TFs) of the receptor cell, Plasma3 cells, in the HC group. C: Ridge plot showing the FC distribution of the downstream target genes (TGs) activated by TFs of Plasma3 cells in the HC group. D: Cell pathways associated with TF activation in receptor cells in the HC group. E: Circos plot of intercellular communication from lymphoid cells in the OR group. F: Sankey diagram describing the L-R pairs and TFs of the receptor cell, bipositive T (BPT) cells, in the OR group. G: Ridge plot showing the fold-change (FC) distribution of the downstream TGs activated by TFs of BPT cells in the OR group (available online).

Supplementary Fig. 12 **Cellcall analysis of lymphoid cells in the severe cases without glucocorticoid therapy (CE) group.** A: Circos plot of intercellular communication from lymphoid cells in the CE group. B: The ligand–receptor (L-R) pairs, the transcription factors (TFs) of the receptor cells CD4T1, and the downstream target genes (TGs) activated by CD4T1 cells in the CE group. C: The L-R pairs, the TFs of the receptor cells CD8T4, and the downstream TGs activated by CD8T4 cells in the CE group. D: The L-R pairs, the TFs of the receptor cells NKT4, and the downstream TGs activated by NKT4 cells in the CE group. E: Cell pathways associated with TF activation in receptor cells in the CE group (available online).

Supplementary Fig. 13 **Cellcall analysis of lymphoid cells in the severe cases receiving glucocorticoid therapy (PT) group.** A: Circos plot of intercellular communication from lymphoid cells in the PT group. B: The ligand-receptor (L-R) pairs, the transcription factors (TFs) of the receptor cells CD4T2, and the downstream target genes (TGs) activated by CD4T2 cells in the PT group. C: The L-R pairs, the TFs of the receptor cells CD8T3, and the downstream TGs activated by CD8T3 cells in the PT group. D: The L-R pairs, the TFs of the receptor cells plasma6, and the downstream TGs activated by plasma6 cells in the PT group. E: Cell pathways associated with TF activation in receptor cells in the PT group (available online).
